# An mHealth Diabetes Intervention for Glucose Control: Health Care Utilization Analysis

**DOI:** 10.2196/10776

**Published:** 2018-10-15

**Authors:** Charlene C Quinn, Krystal K Swasey, Jamila M Torain, Michelle D Shardell, Michael L Terrin, Erik A Barr, Ann L Gruber-Baldini

**Affiliations:** 1 Department of Epidemiology and Public Health University of Maryland School of Medicine Baltimore, MD United States; 2 Translational Gerontology Branch National Institutes on Aging Baltimore, MD United States

**Keywords:** cluster randomized clinical trial, health care, health service utilization, mHealth, type 2 diabetes

## Abstract

**Background:**

Type 2 diabetes (T2D) is a major chronic condition requiring management through lifestyle changes and recommended health service visits. Mobile health (mHealth) is a promising tool to encourage self-management, but few studies have investigated the impact of mHealth on health care utilization.

**Objective:**

The objective of this analysis was to determine the change in 2-year health service utilization and whether utilization explained a 1.9% absolute decrease in glycated hemoglobin (HbA_1c_) over 1-year in the Mobile Diabetes Intervention Study (MDIS).

**Methods:**

We used commercial claims data from 2006 to 2010 linked to enrolled patients’ medical chart data in 26 primary care practices in Maryland, USA. Secondary claims data analyses were available for 56% (92/163) of participants. In the primary MDIS study, physician practices were recruited and randomized to usual care and 1 of 3 increasingly complex interventions. Patients followed physician randomization assignment. The main variables in the analysis included health service utilization by type of service and change in HbA_1c_. The claims data was aggregated into 12 categories of utilization to assess change in 2-year health service usage, comparing rates of usage pre- and posttrial. We also examined whether utilization explained the 1.9% decrease in HbA_1c_ over 1 year in the MDIS cluster randomized clinical trial.

**Results:**

A significant group by time effect was observed in physician office visits, general practitioner visits, other outpatient services, prescription medications, and podiatrist visits. Physician office visits (*P*=.01) and general practitioner visits (*P*=.02) both decreased for all intervention groups during the study period, whereas prescription claims (*P*<.001) increased. The frequency of other outpatient services (*P*=.001) and podiatrist visits (*P*=.04) decreased for the control group and least complex intervention group but increased for the 2 most complex intervention groups. No significant effects of utilization were observed to explain the clinically significant change in HbA_1c_.

**Conclusions:**

Claims data analyses identified patterns of utilization relevant to mHealth interventions. Findings may encourage patients and health providers to discuss the utilization of treatment-recommended services, lab tests, and prescribed medications.

**Trial Registration:**

ClinicalTrials.gov NCT01107015; https://clinicaltrials.gov/ct2/show/NCT01107015 (Archived by Webcite at http://www.webcitation.org/72XgTaxIj)

## Introduction

Type 2 diabetes (T2D) is a major chronic health problem affecting 30.3 million Americans [1]. Persons with uncontrolled diabetes are at increased risk of serious health complications including hypertension, premature death, vision loss, heart disease, stroke, kidney failure, and amputation of toes, feet, or legs [1]. Diabetes is also costly to the US health care system. In the most recent available national study (2012), the total cost of patients diagnosed with diabetes in the United States was US $245 billion, with Medicare paying US $74.3 billion [2]. After adjusting for population sex and age differences, the average medical expenditures of people with diagnosed diabetes were 2.3 times higher than what expenditures would be in the absence of diabetes [2].

Once diagnosed, lifestyle management is the first line of defense for blood glucose (BG) control in diabetes and is continued regardless of prescription medications [3]. Self-management gained through diabetes self-management education and communication with providers is recommended by professional guidelines although studies demonstrate moderate effects on diabetes outcomes. Professional treatment guidelines also recommend BG control through quarterly or annual physician visits with glycated hemoglobin (HbA_1c_) and glucose tests, cardiology and neurology visits, and annual eye and foot exams, as well as multiple health care service utilization to monitor medications, adequacy of individual self-management, and disease progression.

mHealth is a promising tool for delivering interventions designed to promote lifestyle management, but it is neither well understood nor are there well-designed studies of its efficacy and effectiveness. Studies investigating Web-based interventions to promote T2D self-management are inconclusive or demonstrate only moderate effects [4]. Few studies demonstrate even moderate effects that are randomized, include interventions maintaining behavior >6 months, or include older adults or minorities. Although administrative claims data has been used in previous studies to identify the determinants of adherence to diabetes medications and the economic burden of diabetes [5,6], few studies have used administrative claims data to determine changes in health service utilization before and after a mobile phone intervention for adults with T2D. A limited set of studies have demonstrated mixed results of mHealth interventions on health service utilization or costs [7-9].

The analysis reported here, the Mobile Diabetes Intervention Study (MDIS), was a cluster randomized clinical trial (c-RCT) evaluation of a 1-year mobile phone intervention previously described in detail [10,11]. In the c-RCT, mobile phone software allowed patients to securely enter diabetes self-care data on a mobile phone and receive automated real-time educational, behavioral, and motivational messages specific to the patient-entered data. Providers had access to analyzed patient data linked to standards of care and evidence-based guidelines. The 1-year c-RCT reported a 1.9% clinically significant improvement (*P*=.001) in HbA_1c_, the gold standard measure for improved diabetes management [11]. This improvement is important because a reduction of 0.5%-1.0% in HbA_1c_ is considered clinically significant to reduce the risk of comorbid conditions; the FDA recognizes a 0.4% improvement as clinically significant for the prescription antihyperglycemic medications [12-14]. In addition, 5 related substudies evaluating the MDIS impact on physician prescribing behavior, differences by participant age, depression, diabetes distress, and a mixed methods analysis of patient engagement reported modest benefits [15-18]. The purpose of this *a priori*-identified secondary data analysis [10] was to determine whether a mobile phone intervention impacted the utilization of health services identified in commercial insurance administrative claims data and whether changes in health service utilization explained HbA_1c_ change over time. In the current analysis, we hypothesized that the mobile phone personalized behavioral intervention would include more monitoring in-between health service visits and therefore impact utilization and improve HbA_1c_ over a 1-year treatment period.

## Methods

### Design

This study was an administrative claims exploratory data analysis of the previously described c-RCT (NCT01107015) [10,11], where changes in health care utilization during the trial were compared with rates of usage before the trial. Subsequently, the observed changes in health care usage were compared with changes in HbA_1c_. In addition to analyzing claims data, BG data were obtained by abstraction from patients’ medical charts [11]. We planned *a priori* to evaluate utilization but not costs, as cost data would be sparse or skewed in a study of 92 participants [10].

### Participants

The c-RCT was conducted in 26 primary care practices in 4 distinct geographic areas in Maryland, USA [11]. The c-RCT randomly assigned 26 primary care practices to 1 of 3 stepped treatment groups described below (groups 2-4) or a control usual care (UC) group (group 1) [10,11]. Enrolled patients (n=163) followed their physician randomization assignment. The study population included adult patients aged 18-64 years having a physician diagnosis of T2D at least 6 months prior to study enrollment and HbA_1c_≥7.5%. Patients were only eligible for the parent study if their health services were covered by a commercial insurer [10,11]. The primary outcome of the parent study was an absolute change in HbA_1c_ (percentage point of total hemoglobin) comparing the UC (control) group and the maximal treatment group at baseline and 12 months [10,11].

### Intervention

Group 1 received usual care (UC), group 2 received coaching only (CO), group 3 received coaching and patient care provider portal (CPP), and group 4 received coaching and patient care provider portal with decision support (CPDS) [10,11]. The maximal treatment was a mobile phone–based and Web-based self-management patient CPDS [11]. Providers in the CPDS group had access to analyzed patient data linked to the standard of care and evidence-based guidelines, while providers in the CO group received data from their patients if patients chose to share it. Providers in the coaching and patient care provider portal group received unanalyzed patient data [11].

### Measures

For the current secondary data analysis, we analyzed all-cause and diabetes-related adjudicated (paid) claims data for 56% (92/163 participants in the parent study) participants in each study group, (UC, n=28; CO, n=17; CPP, n=13; and CPDS, n=34). All-cause utilization was defined as any claims-based health care utilization inclusive of diabetes and any other diagnosis on the claim. We had access to commercial insurance claims data from 2006 through 2010 for study participants covered by the state’s largest commercial insurer. Other participants were covered by multiple commercial insurers and not included in this analysis. Claims data were aggregated into 12 categories: physician office visits, general practitioner, cardiologists, outpatient services, lab claims, prescription claims, endocrinologists, ophthalmologists, podiatrists, emergency department visits, inpatient visits, and total inpatient hospital days. The physician office visits category was defined as available outpatient records for an office visit validated by the commercial insurance company. General practitioners were defined as any medical visits listed as family practice, general practice, or internal medicine. Category classifications (ie, lab vs prescription claims) were confirmed by the insurer.

Four utilization categories were excluded from the analysis because data were sparse: endocrinologist visits, emergency department visits, inpatient hospital visits, and total inpatient hospital days. Ophthalmologist and podiatrist visits were also infrequent. However, due to their importance in standard diabetes care, we included them in revised models. Data were organized by date and grouped into the 12 months prior to randomization (prerandomization period) and 12 months after randomization (postrandomization period). Patient medical charts were used to collect HbA_1c_ values at baseline and at 3, 6, 9, and 12 months [11].

### Study Oversight

The Institutional Review Board of the University of Maryland, Baltimore, approved this study. A data and safety monitoring board was designated to review the study procedures and adverse events. After enrollment was closed, errors in consent were found and all participants, both physicians and patients, were asked to sign consent forms again, as recommended by the Institutional Review Board. All patients in the final analysis were reconsented.

### Statistical Analysis

Administrative claims data for the participants’ study year were compared with the previous year using generalized linear mixed-effects models to examine the effects of treatment group differences overall, over time, and grouped by time interaction. Specifically, the prerandomization year was the reference year. The treatment effect thus analyzes whether health service visit frequencies were different by group, comparing the year before to the study intervention period. The time by treatment group effect represents the group differential changes from the prerandomization period to the postrandomization period. To address the clustering of physician practices in the parent study, random effects were used to account for within-practice clustering and within-patient correlation.

Due to infrequent ophthalmologist and podiatrist visits (which caused nonconvergence models), these claims’ data were analyzed using repeated measures analysis of variance. Considering the skewed frequency of claims, a Poisson distribution was selected for the outcome.

To examine the impact of utilization on HbA_1c_, general linear models were used. Two tests were conducted for each type of visit: 1 model included only a baseline HbA_1c_ and visit count effect (postrandomization period only) and the other included both of these effects as well as a study group effect. The study group effect examined whether the number of visits, baseline HbA_1c_, and group membership (physician office and general practitioner) predicted 12-month HbA_1c_. The tests without group-effect modeling examined whether the number of visits and baseline HbA_1c_ predicted 12-month HbA_1c_. The statistical software SAS version 9.3 (SAS Institute, Cary, NC) was used for all analyses. The level of significance was set at ≤.05.

## Results

The baseline characteristics for the study population are described in greater detail in the parent study [11]. However, for the purposes of understanding utilization, we report descriptive participant characteristics relevant to utilization outcomes. At study enrollment, the mean age of study participants was 52 years, 54% (50/92) were female and 40% (37/92) were African American individuals (Table 1). A total of 55% (51/92) of patients entered the study with an HbA_1c_ between 7.5% and 8.9 %, although a substantial portion of participants had a baseline HbA_1c_ of >9% (an indication for treatment by an endocrinologist rather than a primary care provider). In the CPDS group, more than half of participants had an HbA_1c_ value of >9%. Most participants had been diagnosed with diabetes for ≥8 years, were nonsmokers, and had at least some college education.

In general, patients were not depressed or distressed by their diabetes, with 79% (73/92) reporting minimal to mild depression and an average diabetes distress scale score of 2.6: <2: little distress and ≥3: high distress [18]. Participants’ total cholesterol levels were desirable, low-density lipoprotein levels were near ideal: UC group: 105.1 (SD 31.8) mg/dl; CO group: 102.3 (SD 25.9) mg/dl; CPP group: 94.6 (SD 30.6) mg/dl; and CPDS group: 112.4 (SD 30.4) mg/dl). Their high-density lipoprotein (HDL) levels were satisfactory: UC group: 44.8 (SD 10.7) mg/dl; CO group: 43.2 (SD 12.5) mg/dl; CPP group: 42.2 (SD 13.0) mg/dl; and CPDS group: 45.9 (SD 11.6) mg/dl.

The baseline characteristics of those not included in our analysis (covered by multiple insurers) were compared with participants insured by the single insurer for whom we had administrative claims data. There were no differences among the 2 groups except for the duration of diabetes. Participants in the administrative claims data group had diabetes longer (mean, 9.2 years) than participants who were not covered by the insurer (mean, 6.9 years, *P*=.02; data not shown).

**Table 1:** Baseline characteristics for participants with commercial insurance coverage (n=92).

Baseline characteristics^a^			UC^b^ (n=28)	CO^c^ (n=17)		CPP^d^ (n=13)		CPDS^e^ (n=34)			*P* value		
**Glycated hemoglobin (%), mean** **(SD)**			9.2 (1.8)	9.5 (2.0)		8.8 (1.6)		9.5 (1.6)			.63		
	7.5-8.9		18 (64.3)	9 (52.9)		8 (61.5)		16 (47.1)			.55		
	≥9.0		10 (35.7)	8 (47.1)		5 (38.5)		18 (52.9)					
Age (years), mean (SD)			52.8 (8.6)	52.4 (9.0)		55.2 (6.4)		52.5 (6.7)			.40		
**Sex, n (%)**										.44			
	Male		10 (36)	10 (59)		7 (54)		15 (44)					
	Female		18 (64)	7 (41)		6 (46)		19 (56)					
**Race, n (%)**										*<.001* ^f^			
	Black (non-Hispanic)		15 (54)	6 (35)		6 (46)		10 (29)					
	White (non-Hispanic)		12 (43)	10 (59)		6 (46)		21 (62)					
Duration of diabetes (years), mean (SD)			10.8 (8.0)	8.4 (5.8)		7.6 (5.3)		9.0 (5.5)			*<.001*		
**Smoking status, n (%)**										*<.001*			
	Nonsmoker		23 (82)	12 (70)		11 (85)		25 (74)					
	Current		4 (14)	4 (24)		2 (15)		3 (9)					
	Former		1 (4)	1 (6)		0 (0)		6 (18)					
**Education, n (%)**										.20			
	High school or trade school		7 (25)	4 (24)		5 (39)		8 (24)					
	Some college or associate’s degree		9 (32)	8 (47)		6 (46)		15 (44)					
	Bachelor’s degree or higher		12 (43)	5 (29)		2 (15)		11 (32)					
**Body mass index (kg/m^2^)^g^, n (%)**										*<.001*			
	Normal		0 (0)	0 (0)		1 (8)		0 (0)					
	Preobese		4 (14)	3 (18)		5 (39)		7 (21)					
	Obese class 1		11 (39)	5 (29)		0 (0)		7 (21)					
	Obese class 2		6 (21)	4 (24)		1 (8)		9 (27)					
	Obese class 3		7 (25)	5 (30)		6 (46)		11 (32)					
**Comorbidities, n (%)**													
	**Hypertension**											.004	
		No	13 (46)	4 (24)		6 (46)		9 (27)					
		Yes	15 (54)	13 (77)		7 (54)		25 (74)					
	**Hypercholesterolemia**											.21	
		No	12 (43)	9 (53)		4 (31)		15 (44)					
		Yes	16 (57)	8 (47)		9 (69)		19 (56)					
	**Coronary artery disease**											*<.001*	
		No	26 (93)	15 (88)		13 (100)		30 (88)					
		Yes	2 (7)		2 (12)		0 (0)		4 (12)				
	**Microvascular complications**											*<.001*	
		No	24 (86)		16 (94)		12 (92)		30 (88)				
		Yes	4 (14)	1 (6)		1 (8)		4 (12)					
	**Depression (PHQ-9^h^) score**											.21	
		Minimal to mild (0-9)	22 (30)	15 (21)		10 (14)		26 (35)					
		Moderate (10-14)	2 (20)	0 (0)		2 (20)		6 (60)					
		Moderately severe (15-19)	4 (57)	2 (29)		1 (14)		0 (0)					
		Severe depression (20-27)	0 (0)	0 (0)		0 (0)		2 (100.0)					
**Patient-reported outcomes, mean (SD)**													
	Diabetes Distress Scale^i^		2.4 (0.8)	2.6 (0.9)		2.7 (0.7)		2.8 (1.0)					.41
	Diabetes symptom inventory^j^		20.7 (15.0)	18.1 (13.8)		23.3 (17.1)		23.8 (16.8)					.65
**Laboratory outcomes, mean (SD)**													
	Systolic blood pressure (mmHg)		133.3 (25.1)	130.9 (17.7)		134.8 (14.4)		130.2 (12.2)					.84
	Diastolic blood pressure (mmHg)		78.9 (13.1)	79.7 (11.5)		81.2 (7.0)		78.3 (8.1)					.85
	Low-density lipoprotein (mg/dL)		105.1 (31.8)	102.3 (25.9)		94.6 (30.6)		112.4 (30.4)					.32
	High-density lipoprotein (mg/dL)		44.8 (10.7)	43.2 (12.5)		42.2 (13.0)		45.9 (11.6)					.76
	Triglycerides (mg/dL)		191.6 (193.0)	161.7 (101.4)		168.9 (116.8)		173.9 (120.8)					.91
	Total cholesterol (mg/dL)		188.3 (56.7)	179.2 (23.8)		167.4 (43.3)		191.0 (31.6)					.35

^a^Data Source: claims data, primary care provider office patient medical records, and research surveys.

^b^UC: usual care.

^c^CO: coaching only.

^d^CPP: coaching and patient care provider portal.

^e^CPDS: coaching and patient care provider portal with decision support.

^f^All italicized values indicate statistical significance, *P*<.05

^g^BMI: normal, 18.5-24.9 kg/m^2^; preobese, 25-29.9 kg/m^2^; obese class 1, 30-34.9 kg/m^2^; obese class 2, 35-39.9 kg/m^2^; obese class 3, ≥40.0 kg/m^2^.

^h^PHQ-9: Patient Health Questionnaire-9.

^i^17-item measure, a mean across 17 items; each item scored from 1 (little distress) to 6 (serious distress).

^j^9-item measure, mean scores range from 0 (no dysfunction) to 100 (worst possible health status).

Table 2 compares changes in utilization from the previous year to the study year by service type. Physician office visits (*P*=.01), general practitioner visits (*P*=.02), other outpatient services (*P*=.001), prescription claims (*P*<.001), and podiatrist visits (*P*=.04) showed significant changes in utilization. Physician office visits and general practitioner visits both decreased over time while prescription claims increased. Interestingly, UC and CO groups experienced decreases in the utilization of other outpatient services and for podiatrist visits, whereas groups CPP and CPDS increased their utilization of these services.

A significant group by time effect was observed in physician office visits, general practitioner visits, other outpatient services, and prescription claims. The CPDS group, which had the most intense intervention, had the smallest decline of all treatment groups for physician office visits (−2.68), while the UC group had the largest decline in physician office visits (−5.09; *P*=.01). Both groups had similar physician office visit counts for the year prior to the study, but the CPDS group maintained higher utilization during the study period. For general practitioner visits, the CPDS group showed the smallest decrease in visits (−1.38), while the CPP group showed the largest decrease in visits (−3.39; *P*=.02). The groups had similar visit counts for the year prior to the study, but the CPDS group maintained a higher utilization during the study period than the other groups. In other outpatient services, the CPDS group had a significant increase in the number of claims in the study year (+0.35), while the UC group had a significant decline (−3.38; *P*=.001). This may be partially due to the unequal visit counts of the prior year, as the UC group had the highest prior year other outpatient services visit count. The CPDS group also had a significant increase in prescription claims from the prior year to the study year (+43.95) compared with the CPP group, which showed the only decline in prescription claims (−2.19; *P*<.001). Repeated measures analysis of variance revealed that podiatrist claim changes from the prior year to the study year were different across the groups. The CPDS group showed the greatest gain in podiatry visits (+0.48), while the CO group showed the greatest decline (−0.88; *P*=.04). Group by time effects for ophthalmologists, cardiologists, or lab claims were not significant.

Changes in service utilization over the study period had no significant effect on HbA_1c_ (Table 3). No significant effects of the various study year utilization visit counts were observed on changes in 12-month HbA_1c_ to explain the clinically significant results obtained in the parent study [11].

**Table 2:** Changes in service utilization by type of service, by time (1 year) and group difference (n=92).

Claim type^a^ and visits		UC^b^ (n=28), mean (SD)	CO^c^ (n=17), mean (SD)	CPP^d^ (n=13), mean (SD)	CPDS^e^ (n=34), mean (SD)	Group time, *P* value
**Physician office visits**						
	Previous year	10.00 (6.74)	10.00 (7.81)	7.79 (7.83)	10.45 (7.06)	—
	Study year	4.91 (4.43)	6.20 (5.36)	4.08 (4.98)	7.76 (5.35)	—
	Increment	−5.09 (5.77)	−3.75 (6.64)	−4.11 (4.22)	−2.68 (5.91)	*.01* ^*f*^
**General practitioner visits**						
	Previous year	5.29 (3.63)	4.75 (3.21)	5.36 (5.18)	5.72 (4.09)	—
	Study year	2.44 (2.62)	2.60 (2.40)	2.33 (2.61)	4.34 (3.62)	—
	Increment	−2.84 (3.95)	−2.19 (2.37)	−3.39 (3.95)	−1.38 (2.74)	*.02*
**Cardiologist visits**						
	Previous year	0.40 (1.09)	0.69 (2.27)	0.31 (1.11)	0.44 (1.05)	—
	Study year	0.14 (0.59)	0.53 (1.12)	0.17 (0.39)	0.41 (1.02)	—
	Increment	−0.26 (0.62)	−0.13 (2.22)	−0.17 (0.94)	−0.03 (1.29)	.54
**Other outpatient services**						
	Previous year	6.99 (12.71)	2.00 (3.18)	2.49 (7.17)	3.93 (7.47)	—
	Study year	3.61 (5.28)	1.00 (1.54)	2.92 (4.17)	4.27 (8.32)	—
	Increment	−3.38 (10.34)	−0.94 (3.17)	+0.22 (2.64)	+0.35 (7.58)	*.001*
**Lab claims**						
	Previous year	4.17 (3.71)	3.31 (2.18)	2.77 (2.23)	4.61 (2.96)	—
	Study year	2.15 (2.00)	2.30 (2.66)	1.67 (1.61)	4.20 (3.27)	—
	Increment	−2.02 (3.70)	−0.94 (3.28)	−1.17 (1.98)	−0.41 (2.96)	.09
**Prescription claims**						
	Previous year	11.88 (32.80)	3.19 (8.01)	25.95 (76.71)	7.97 (19.93)	—
	Study year	19.28 (48.14)	29.03 (51.10)	25.92 (60.94)	50.93 (102.04)	—
	Increment	+7.39 (20.90)	+15.25 (25.80)	−2.19 (38.51)	+42.95 (84.66)	*<.001*
**Ophthalmologist visit**						
	Previous year	0.11 (0.42)	0 (0)	0.08 (0.28)	0.12 (0.33)	—
	Study year	0.18 (0.77)	0 (0)	0 (0)	0.15 (0.50)	—
	Increment	+0.07 (0.47)	0 (0)	−0.08 (0.29)	+0.03 (0.39)	.67
**Podiatrist visit**						
	Previous year	0.57 (1.43)	1.50 (2.88)	0 (0)	0.37 (1.2)	—
	Study year	0.46 (1.23)	0.59 (1.58)	0.08 (0.29)	0.85 (1.44)	—
	Increment	−0.11 (1.20)	−0.88 (2.13)	+0.08 (0.29)	+0.48 (1.67)	*.04*

^a^Data source: claims data.

^b^UC: usual care.

^c^CO: coaching only.

^d^CPP: coaching and patient care provider portal.

^e^CPDS: coaching and patient care provider portal with decision support.

^f^All italicized values indicate statistical significance, *P*<.05

**Table 3:** Mobile Diabetes Intervention Study: Changes in utilization by service type and effect on HbA_1c_ over a 1-year period.

Claim type^a^	Claims during study, mean (SD)	HbA_1c_^b^ change per visit			
		Model without group effect			Model with group effect	
		Estimate	*P* value		Estimate	*P* value
Physician office visits	6.1 (5.2)	−0.016	.61		−0.01	.75
General practitioner visits	3.2 (3.1)	0.002	.97		0.009	.86
Cardiologist visits	0.3 (0.9)	−0.026	.87		0.024	.88
Ophthalmologist visits	0.1 (0.5)	0.083	.76		−0.012	.96
Podiatrist visits	0.6 (1.3)	−0.185	.09		−0.188	.08
Outpatient services	3.3 (6.1)	0.002	.96		−0.009	.75
Lab claims	2.9 (2.8)	−0.009	.87		0.001	.99
Prescription claims	33.8 (75.0)	−0.001	.51		−0.001	.61

^a^Data Source: Commercial Insurer, November 2006-January 2010.

^b^HbA_1c_: glycated hemoglobin.

## Discussion

### Principal Findings

To our knowledge, this administrative claims analysis is the first study assessing a c-RCT mobile phone diabetes intervention’s impact on health service utilization. This study expands on the study by Quinn et al [11] by assessing the intervention’s effect on changes in health service utilization and whether those changes explain the clinically significant change in HbA_1c_ reported in the primary study. We found that mobile phone-based treatment and behavioral coaching intervention decreased physician office visits, general practitioner visits, and lab claims over time while prescription claims increased. The issue of whether an increase or decrease in claims as a result of the intervention is desirable or undesirable is unclear. Increases in medication prescriptions may be a good outcome, suggesting that appropriate physician intensification occurred and participants were more appropriately taking their medications [17]. Decreases in physician visits and labs saves money but could either indicate less need for care, or if too infrequent, may suggest mHealth participants are inappropriately substituting the in-person care with attention from the intervention.

Our finding of no significant effects of utilization visits to explain the change in 12-month HbA_1c_ obtained in the parent study [11] may be due to the intervention’s multiple behavior change strategies. In a follow-up mixed methods analysis of patient engagement in the parent study, we learned that some patient behaviors (glucose monitoring, healthy eating, and taking medication) contributed to greater changes in HbA_1c_ [15]. It may also be that our sample of claims data was too small or too short in follow-up to evaluate the impact of utilization on changes in HbA_1c_ over a 1-year period.

The generally positive findings reported here need to be put into context with the mixed economic results from diabetes management interventions reported in other studies. Nundy examined the impact of a 6-month mHealth demonstration project among adults (n=74) with type 1 and type 2 diabetes who were members of an academic medical center’s employee health plan. Although those authors observed pre-post improvements in glycemic control (*P*=.01) and a significant decrease in per person outpatient visits, only 20% of the eligible population participated in the study [19]. Another study found improvements in clinical measures but no impact on health care utilization or cost [20]. None of these studies are directly analogous to this study, which highlights the difficulty in determining successful integration of mobile phone diabetes interventions in clinical settings as a reimbursable service.

### Payers

The results of our study will inform payers attempting to understand the potential of mobile phone diabetes management technology. Payers have been reluctant to reimburse for mHealth visits, partly due to lack of evidence that mHealth interventions make a difference in utilization and related costs. Payers’ views are short term, not long term, because members may not be enrolled in their plan for the next insured year. The prevention or delay of complications require years. Therefore, a short-term outcome, such as change in HbA_1c_ as demonstrated in our primary study, as an indicator of prevention may be more appealing to payers than specific utilization effectiveness of an mHealth intervention.

Payers are “experimenting” with health and wellness apps, but current reimbursement payments for mHealth care are largely limited to remote rural areas. Payers have a financial interest in minimizing their risk by actively promoting the health of their policyholders. However, most health care technologies are used in the current fee-for-service (FFS) model, including our evaluation [21]. In a FFS system in which there are only codes for medical devices and human to clinician visits, “there are few, if any, reimbursement codes that exist for frequent high-value patient touchpoints driven by technology rather than humans.” [22]. The potential benefits of mobile phone diabetes care from the perspective of payers may be driven by the transformation of the incentives from pay for service to pay-for-value or performance. Mobile phone diabetes management, such as our intervention focusing on behavioral change based on digital contacts between patients and providers that improves clinical outcomes and utilization metrics, is well-placed in a bundled payment model. The bundled payment model could include a per-patient amount for a bundle of services to be provided, preferably with payment based on agreed-upon clinical outcomes achieved by the mobile phone digital contacts, instead of an FFS payment system based solely on the number of office contacts.

### Limitations

We advise caution in generalizing our findings. Participants in the study were insured by a single commercial insurer and may have experience with and access to resources including individual group practice guidelines, access to specialists, and variations in insurance plan coverage and coinsurance different from the rest of the population with T2D. We attempted to address these differences by enrolling multiple community physicians to participate in the study and randomization at the practice level with patient enrollment following physician randomization assignment. Administrative claims data may not adequately capture service utilization by persons with T2D. For example, diabetes is infrequently the primary diagnosis for emergency department visits or hospitalizations but is often an underlying condition (eg, to myocardial infarction). The analysis was unlikely to see more severe conditions requiring hospitalizations because of exclusion criteria and therefore unlikely to observe high utilizers where diabetes is severe and uncontrolled, (eg, gangrene or kidney failure), although 45% (41/92) of participants had HbA_1c_>9% at enrollment. Improvements in utilization, both increases and decreases depending on the health service, may have occurred after the utilization analytic year. Some utilization was required of the study treatment (ie, visiting primary care provider and receiving prescription for HbA_1c_ tests).

### Conclusion

Our program of studies [11,15,17,18,23], including the analysis reported here, demonstrates that a mobile phone diabetes technology achieved a clinically significant change in BG control and that important service utilization increased (pharmacy) or decreased (physician office visits). These findings may help persons with T2D diabetes engage with health service providers and participate in decisions to receive services, lab tests, and prescribed medications recommended by treatment guidelines.

## Abbreviations

BG: blood glucose
CO: coaching only
CPDS: coaching and patient care provider portal with decision support
CPP: coaching and patient care provider portal group
c-RCT: cluster randomized clinical trial
FFS: fee-for-service
BG: blood glucose
HbA_1c_: glycated hemoglobin
MDIS: Mobile Phone Diabetes Intervention Study
T2D: type 2 diabetes
UC: usual care
